# Mast Cell Activation and Microtubule Organization Are Modulated by Miltefosine Through Protein Kinase C Inhibition

**DOI:** 10.3389/fimmu.2018.01563

**Published:** 2018-07-09

**Authors:** Zuzana Rubíková, Vadym Sulimenko, Tomáš Paulenda, Pavel Dráber

**Affiliations:** ^1^Department of Biology of Cytoskeleton, Institute of Molecular Genetics, Czech Academy of Sciences, Prague, Czechia; ^2^Department of Signal Transduction, Institute of Molecular Genetics, Czech Academy of Sciences, Prague, Czechia

**Keywords:** bone marrow-derived mast cells, cell activation, microtubules, miltefosine, protein kinase C

## Abstract

Mast cells play an effector role in innate immunity, allergy, and inflammation. Antigen-mediated activation of mast cells initiates signaling events leading to Ca^2+^ response and the release of inflammatory and allergic mediators from granules. Diseases associated with deregulated mast cell functions are hard to treat and there is an increasing demand for new therapeutic strategies. Miltefosine (hexadecylphosphocholine) is a new candidate for treatment of mast cell-driven diseases as it inhibits activation of mast cells. It has been proposed that miltefosine acts as a lipid raft modulator through its interference with the structural organization of surface receptors in the cell membrane. However, molecular mechanisms of its action are not fully understood. Here, we report that in antigen-activated bone marrow-derived mast cells (BMMCs), miltefosine inhibits degranulation, reorganization of microtubules, as well as antigen-induced chemotaxis. While aggregation and tyrosine phosphorylation of IgE receptors were suppressed in activated cells pre-treated with miltefosine, overall tyrosine phosphorylation levels of Lyn and Syk kinases, and Ca^2+^ influx were not inhibited. In contrast, lipid raft disruptor methyl-β-cyclodextrin attenuated the Ca^2+^ influx. Tagged-miltefosine rapidly localized into the cell interior, and live-cell imaging of BMMCs with labeled intracellular granules disclosed that miltefosine inhibited movement of some granules. Immunoprecipitation and *in vitro* kinase assays revealed that miltefosine inhibited Ca^2+^- and diacylglycerol-regulated conventional protein kinase C (cPKC) isoforms that are important for mast cell degranulation. Inhibition of cPKCs by specific inhibitor Ly333531 affected activation of BMMCs in the same way as miltefosine. Collectively, our data suggest that miltefosine modulates mast cells both at the plasma membrane and in the cytosol by inhibition of cPKCs. This alters intracellular signaling pathway(s) directed to microtubules, degranulation, and migration.

## Introduction

Mast cells play a pivotal role in innate immunity, allergy, and inflammation. Diseases associated with deregulated mast cell functions are hard to treat, and so the demand for new and better treatments targeting mast cell activation pathways increases. Mast cells express on their surfaces receptors with a high affinity for IgE (FcεRIs). Aggregation of FcεRIs by multivalent antigen (Ag)-IgE complexes leads to activation of signaling pathways resulting in the release of Ca^2+^ from the endoplasmic reticulum (ER) and subsequent activation of store-operated Ca^2+^ entry (SOCE). The influx of free Ca^2+^ is important for replenishment of Ca^2+^ in ER, but also works as a second messenger for further signaling. Activation events result in the release of preformed granule mediators and *de novo* synthesis and secretion of bioactive compounds, including lipid mediators, cytokines, and chemokines (1). Besides that, mast cell activation by FcεRI aggregation is accompanied with changes in cell morphology, enhanced adhesion, and migration. It was reported that activation of mast cells induces increased formation of microtubules (2, 3) and their reorganization into protrusions containing microtubules (microtubule protrusions) (4, 5). Independent of FcεRI aggregation, the activation events can be mimicked by non-specific activators, such as protein tyrosine phosphatase inhibitor pervanadate, inhibitor of ER Ca^2+^-ATPase pumps thapsigargin (4), or calcium ionophore A23187 (6).

A promising candidate for novel therapeutic strategies in mast cell-driven diseases is miltefosine (hexadecylphosphocholine), as it inhibits activation in human mast cells (7) and reduces disease progression in patients with mast cell-derived mastocytosis (8), urticaria (9), and atopic dermatitis (10). Moreover, miltefosine is used as a treatment of leishmaniasis (11) and free-living amebae infections (12).

Miltefosine is a derivative of plasmalogen phospholipids (13), which is taken up by cells in a lipid raft-dependent manner (14). It has been proposed that miltefosine acts as a lipid raft modulator through its interference with the structural organization of surface receptors in the cell membrane (15). Besides that, it modulates different signaling pathways. It has been reported that miltefosine affects phosphatidylcholine synthesis and stress-activated protein kinase/Jun N-terminal kinase apoptotic pathway (16), phosphatidylinositol 3-kinase (PI3K)/Akt survival pathway (17), as well as the activity of phospholipase Cβ (18), phospholipase D (19), and protein kinase C (PKC) (20). Despite this knowledge, the molecular mechanisms of miltefosine action in mast cells remain poorly understood.

To get deeper insight into the function(s) of miltefosine in mast cells we evaluated early stages of cell activation after crosslinking of FcεRIs, Ca^2+^ influx, degranulation, microtubule reorganization, and migration in bone marrow-derived mast cells (BMMCs) treated with miltefosine. Moreover, we localized miltefosine in BMMCs and evaluated its effect on intracellular granule movement. Our results indicate that miltefosine does not regulate mast cells only through lipid raft modulation, but also by inhibition of Ca^2+^-dependent PKCs affecting cytosolic signaling pathways that modulate microtubule organization, degranulation, and migration of mast cells.

## Materials and Methods

### Reagents

Calcium ionophore A23187, dinitrophenyl-albumin (DNP-albumin), fibronectin, Ly333531, methyl-β-cyclodextrin (MβCD), miltefosine, probenecid, puromycin, thapsigargin, Trypan blue, and 4-nitrophenyl N-acetyl-β-D-glucosaminide (4-NAG) were from Sigma-Aldrich (St. Louis, MO, USA). Fura-2-acetoxymetyl ester (Fura-2-AM) was purchased from Invitrogen (Carlsbad, CA, USA). Collagen I was from Advanced BioMatrix (San Diego, CA, USA). Protein A Sepharose™ CL-4B was from GE Healthcare Life Sciences (Chicago, IL, USA) and SuperSignal WestPico Chemiluminescent reagent was from Pierce (Rockford, IL, USA). Wheat germ agglutinin (WGA) conjugated with Alexa Fluor 555 (WGA-AF555) was purchased from Molecular Probes (Eugene, OR, USA).

### Antibodies

Mouse monoclonal antibody (mAb) TUB 2.1 (IgG1) to β-tubulin conjugated with indocarbocyanate (Cy3), mouse mAb SPE-7 (IgE) specific for DNP, and mouse mAb PY-20 (IgG2b) to phosphotyrosine were from Sigma-Aldrich (St. Louis, MO, USA). α-Tubulin was detected with rabbit Ab (GTX15246) from Genetex (Irvine, CA, USA). Rabbit polyclonal Ab to mouse IgE was described previously (21) and rabbit mAb to PKCαβγ was from Abcam (Cambridge, UK). Mouse mAb SKB1 (IgG) to Akt and mouse mAb 4G10 (IgG2b) to phosphotyrosine conjugated with horseradish peroxidase (HRP) were from Upstate Laboratories (Syracuse, NY, USA). Rabbit polyclonal Abs to Lyn (Lyn44), Syk (N-19), and phospho-Akt (Ser^473^) were from Santa Cruz Biotechnology (Dallas, TX, USA). Rabbit Ab to phospho-Akt (Thr^308^) was from Cell Signaling (Danvers, MA, USA). Preparation of rabbit Ab to LAT and mouse mAb LAT.1D1 (IgG2a) to LAT were described previously (22, 23). Mouse mAb TU-32 (IgG1) to γ-tubulin was described previously (24). Anti-mouse and anti-rabbit Abs conjugated with HRP were from Promega Biotec (Madison, WI, USA). Anti-mouse Ab conjugated with DyLight549 was from Jackson ImmunoResearch Laboratories (West Grove, PA, USA).

### Cell Cultures and Activation

Primary culture of bone marrow-derived mast cells from BALB/c and cells of mouse BMMC lines (25) were prepared and cultured as previously described (4). For immunofluorescence experiments, cells were overlaid on fibronectin-coated coverslips (4). Cells were only sensitized with DNP-specific IgE (mouse mAb SPE-7; 1 µg/ml) for 4 h in culture medium without 10% WEHI-3 cell supernatant and activated with Ag (DNP-albumin conjugate; 1 µg/ml; 30–40 mol DNP/mol albumin) for 3 min in culture medium with 0.5% FCS and without 10% WEHI-3 cell supernatant (activation medium) (4). Alternatively, sensitized cells were activated by crosslinking of bound IgE with anti-mouse Ab conjugated with DyLight549 (1.5 µg/ml) for 20 min at 37°C as described previously (26). Cells were also activated for 15 min at 37°C in activation medium containing 2 µM thapsigargin or pervanadate as described previously (4) or 0.5 µM ionophore A23187. Control human retinal pigment epithelial cells hTERT-RPE1 (RPE1) (Dr. M. Bonhivers, Université Bordeaux, Bordeaux, France) were cultured as described previously (27).

Cells were pre-treated with miltefosine at final concentration 5–25 µM for 15–60 min at 37°C prior to activation. In some cases, cells were incubated with 1–15 µM BODIPY-labeled miltefosine (MT-11c-6EtBDPY) (28) for 1–60 min at 37°C. If not specified otherwise, cells were pre-treated with miltefosine or BODIPY-miltefosine at final concentration of 15 µM for 15 min, and compounds were also present in the course of activation. Alternatively, cells were incubated under the same conditions with 0.015–10 mM MβCD or 0.5–15 µM Ly333531.

Trypan blue exclusion test was used to evaluate the effect of miltefosine treatment on viability of BMMCs.

### Reverse Transcription PCR

Total RNAs from BMMCs or mouse brain were isolated by the RNeasy Mini kit (QIAGEN, Valencia, CA, USA) and converted to cDNAs using the SuperScript^®^ VILO cDNA Synthesis Kit (Thermo Fisher Scientific, Waltham, MA, USA) according to the manufacturer’s protocol. PCRs were performed with primers specific for mouse PKCα (*Prkca*, NM_011101.3; forward 5′-GTCTCAGAGCTAATGAAGATG-3′ and reverse 5′-TTGGCTTTCTCAAACTTCTG-3′), PKCβ (*Prkcb*, NM_008855.2, and NM_001316672.1; primers anneal to all transcript variants; forward 5′-GAATCAGACAAAGACAGAAGAC-3′ and reverse 5′-CTTAGTAACTTGAACCAGCC-3′), and PKCγ (*Prkcg*; NM_011102.4, NM_001291434.1; primers anneal to all transcript variants; forward 5′-AATGTACCGGTGGCCGATGCT-3′; and reverse 5′-AGGCGGTCCGGAGTCTGAAA-3′). Mouse actin (*Actb*; NM_007393; forward 5′-GGACCTGACGGACTACCTCATG-3′ and reverse 5′-TCTTTGATGTCACGCACGATTT-3′) was used as house-keeping gene. All primers (Sigma-Aldrich) were tested *in silico* by NCBI BLAST to amplify specific targets. The PCR efficiencies for tested PKC isoforms were similar. Quantitative PCRs were performed in the LightCycler 480 System (Roche, Mannheim, Germany) as described previously (29). Amplified fragments were separated on 2% agarose gel and stained by GelRed Nucleic Acid Gel Stain (Biotium, Fremont, CA, USA).

### Determination of Intracellular Ca^2+^ Concentrations

Changes in the level of free intracellular Ca^2+^ were measured using Fura-2-AM as a cell permeant calcium reporter following protocol for sample handling as described in Ref. (4). Intracellular free Ca^2+^ was measured in microplate reader Infinite M200 (Tecan, Männedorf, Switzerland) as a ratio of Fura emissions at 510 nm after excitation with 340 and 380 nm (340/380) lasers at the indicated time points. After measurement of the Ca^2+^ basic level, activation was triggered by addition of Ag, thapsigargin, or ionophore A23187.

### Degranulation Assay

The degree of degranulation was quantified as the release of β-hexosaminidase from Ag-, thapsigargin-, pervanadate-, or ionophore-activated cells, using 4-NAG as substrate (2). The extent of degranulation was calculated as follows: absorbance of culture supernatant × 100/absorbance of total cell lysate and normalized to control cells.

### Immunoprecipitations, Kinase Assay, and Immunoblotting

For immunoprecipitation experiments, BMMCs (1 × 10^7^ cells per reaction) were activated with Ag or thapsigargin. Immunoprecipitation was performed as previously described (30). Cell extracts were incubated with protein A beads saturated with (i) Ab to IgE; (ii) Ab to PKCαβγ; (iii) Ab to Lyn; (iv) Ab to Syk; (v) Ab to LAT; or (vi) immobilized protein A alone. *In vitro* kinase assay was essentially performed as described and the ^32^P-labeled proteins were detected by autoradiography using the Amersham Typhoon scanner (GE Healthcare Europe GmbH, Freiburg, Germany). Whole cell extract preparation, gel electrophoresis, and immunoblotting were described elsewhere (4). Abs to γ-tubulin (in the form of spent culture supernatant) and PKCαβγ were diluted 1:2 and 1:3,000, respectively. Abs to Lyn, Syk, LAT (mAb LAT1.D1), Akt, phospho-Akt (Thr^308^), and phospho-Akt (Ser^473^) were diluted 1:2,000, 1:2,000, 1:2,000, 1:2,000, 1:500, and 1:1,000, respectively. Phosphotyrosine was detected by anti-phosphotyrosine mAb 4G10 conjugated with HRP (dilution 1:2,000) or by mAb PY-20 (dilution 1:2,000). Bound primary antibodies were detected after incubation of the blots with HRP-conjugated secondary Abs diluted 1:10,000. HRP signal was detected with chemiluminescent reagents and the LAS 3000 imaging system (Fujifilm, Düsseldorf, Germany). AIDA image analyzer (ver.4) software (Raytest, Straubenhardt, Germany) was used for quantification of signals from autoradiographs and immunoblots.

### Chemotaxis and Cell Migration Assay

Chemotaxis and cell migration assays were performed in μ-Slide Chemotaxis^3D^ chambers according to the protocols described elsewhere (31). In the case of chemotaxis assay, BMMCs were sensitized prior to seeding and one reservoir of each chamber was supplied with Ag at concentration 100 ng/ml. In miltefosine-, MβCD-, or Ly333531-treated cells, the drug was added to collagen I gel and to reservoirs and was present during the gel polymerization and imaging.

### Immunofluorescence Microscopy

Immunofluorescence microscopy was performed with cells attached to fibronectin-coated coverslips fixed as described (32). TUB 2.1 mAb conjugated with Cy3 was diluted 1:600. DyLight549-conjugated anti-mouse Ab was diluted 1:500. To visualize FcεRI aggregation, cells were fixed without Triton X-100 extraction to preserve intact cell membranes. The preparations were examined with an Olympus A70 Provis microscope (Olympus, Hamburg, Germany) or in the Delta Vision Core system (Applied Precision, Issaquah, WA, USA). The conjugated secondary Ab did not give any detectable staining. Live cell imaging of BMMCs labeled with 15 µM BODIPY-miltefosine was performed in the Delta Vision Core system. Images were deconvolved by integrated deconvolution software. Determination of the number of cells that responded to activation events by generation of microtubule protrusions was done as described previously. Three experiments were performed, and in each experiment 300–500 cells were examined (4).

### Time-Lapse Imaging

For chemotaxis and cell migration assay, cells were imaged with a Leica DMI6000 inverted microscope at 37°C and 5% CO_2_. Time-lapse sequences of bright-field images were taken for 7 (chemotaxis) or 3 h (cell migration), at 1 min intervals with the intensity of 71 and exposure time 6 ms. To evaluate the intracellular granule movement, cells were incubated with WGA-AF555 (5 µg/ml) for 10 min and time-lapse sequences were acquired in the Delta Vision Core System.

### Image Analysis

For analysis of the granule movement, time-lapses were registered by StackReg in ImageJ and processed using ImageJ Kymograph plugin. Fluorescence intensity of FcεRI aggregation was quantified using ImageJ based on the mean pixel intensity of each cell.

Chemotactic response and cell migration were analyzed from time-lapse movies as described previously (31). Cells were tracked in the MetaMorph program using the Track Object application. The data were processed by Chemotaxis and Migration Tool in ImageJ. Analysis was performed for 7 and 3 h imaging.

### Statistical Analysis

All data are presented as mean ± SD or SE, as indicated. For statistical analysis, the two-tailed, unpaired Student’s *t*-test was applied.

## Results

### Degranulation in Activated BMMCs Is Inhibited by Miltefosine

**Figure 1:**
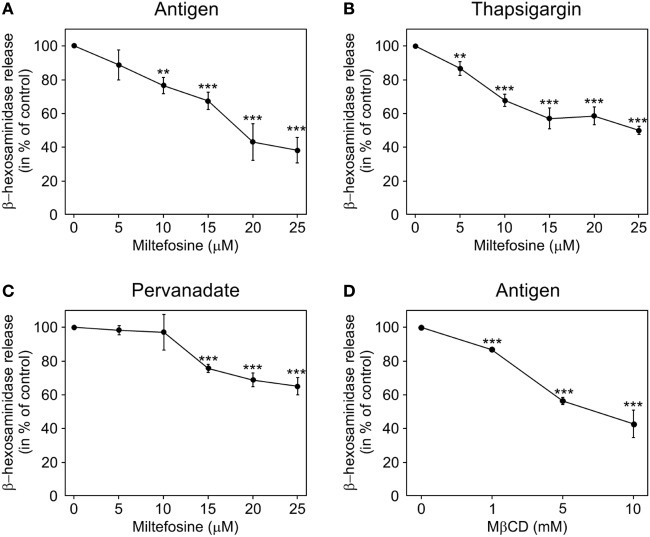
Miltefosine and methyl-β-cyclodextrin (MβCD) inhibit degranulation in activated bone marrow-derived mast cells (BMMCs). BMMCs pre-incubated with different concentrations of miltefosine (5–25 µM) **(A–C)** or MβCD (1–10 mM) **(D)** were activated and degranulation was measured by β-hexosaminidase release. **(A,D)** IgE-sensitized cells were activated by high affinity IgE receptor aggregation with Ag. **(B)** Cells activated with thapsigargin. **(C)** Cells activated with pervanadate. Values represent mean ± SD (*n* = 3); **p < 0.01 and ***p < 0.001.

Miltefosine is known to inhibit mediator release in human mast cells (7). To test whether the same holds true for mouse BMMCs, the degree of degranulation in miltefosine pre-treated and activated BMMCs was determined. Cells were incubated with different concentrations of miltefosine in the range of 5–25 µM for 15 min. The release of β-hexosaminidase in cells activated by FcεRI aggregation (Figure 1A) decreased in a miltefosine-dose-dependent manner. Alternatively, BMMCs were activated by thapsigargin. Miltefosine decreased the level of degranulation in a dose-dependent manner as well (Figure 1B). A less prominent inhibitory effect was observed after stimulation of BMMCs by pervanadate (Figure 1C). The least effect (~21% inhibition at miltefosine concentration 25 µM) was detected when the cells were activated by calcium ionophore A23187 (data not shown). As miltefosine was proposed to act as a lipid raft modulator (15), we treated BMMCs with MβCD, a typical lipid raft disruptor. MβCD inhibited β-hexosaminidase release in cells activated by FcεRI aggregation (Figure 1D) at substantially higher concentrations. The maximal inhibitory effect (~60% inhibition) was observed at MβCD concentration of 10 mM.

To evaluate whether inhibitory effect of miltefosine is due to changes in cell viability, we performed Trypan blue exclusion test. Viability of BMMCs treated with 15 µM miltefosine for 15, 30, and 60 min in activation medium was 94.7 ± 7; 94.6 ± 7, and 95.0 ± 7% (mean ± SD; *n* = 3), respectively. As viability of BMMCs treated with 25 µM miltefosine fell to ~50%, miltefosine was used at a maximal concentration of 15 µM in the following experiments.

Taken collectively, miltefosine inhibits degranulation in mouse BMMCs activated either specifically by FcεRI aggregation, or unspecifically by thapsigargin, pervanadate, or calcium ionophore.

### Miltefosine Modulates Microtubule Organization and Cell Migration in BMMCs

**Figure 2:**
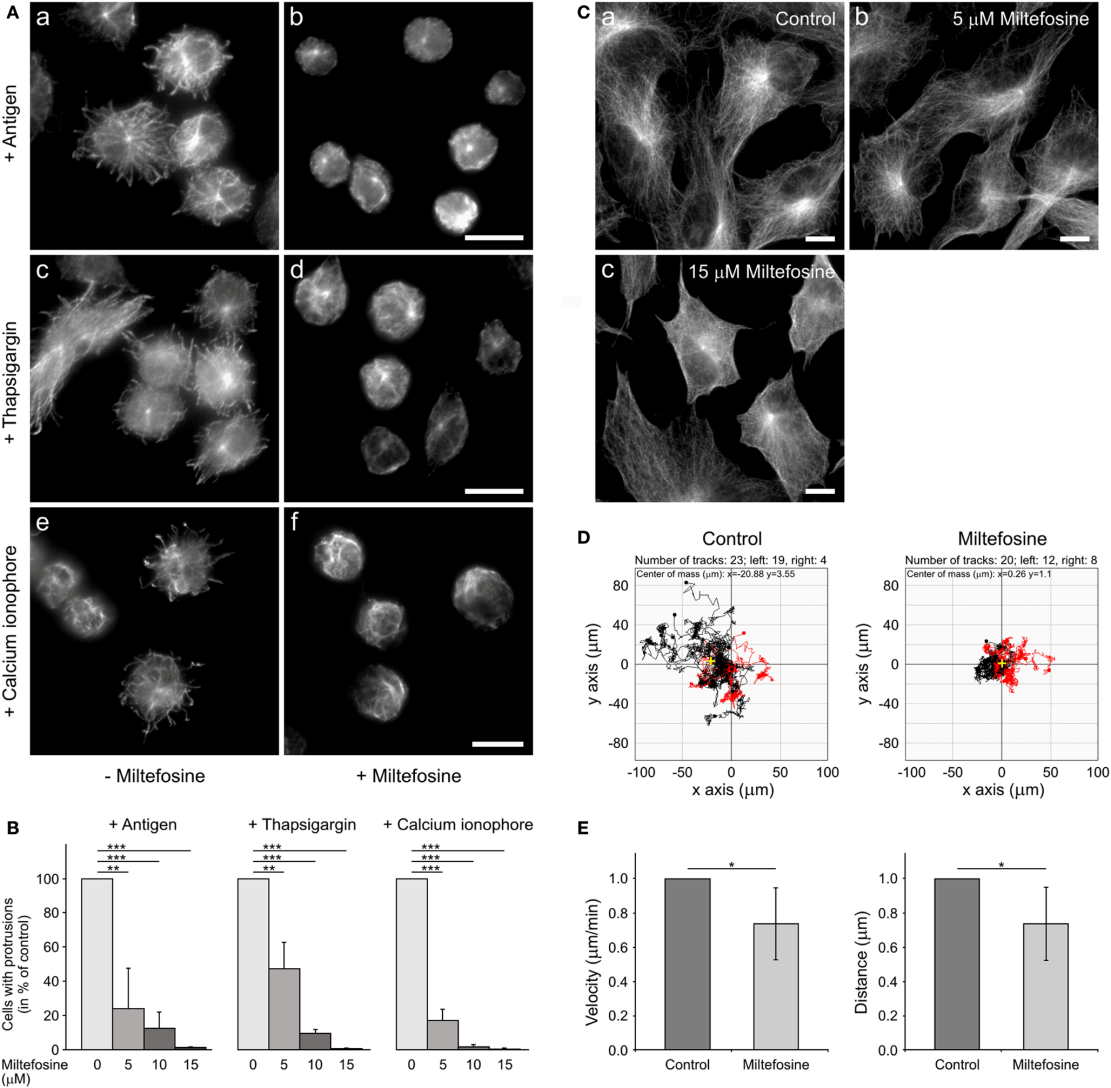
Microtubule organization in activated bone marrow-derived mast cells (BMMCs) and Ag-induced chemotaxis are affected by miltefosine. **(A)** Microtubule organization. BMMCs incubated in the absence (a,c,e) or presence (b,d,f) of 15 µM miltefosine were activated by high affinity IgE receptor (FcεRI) aggregation (a,b; antigen), thapsigargin (c,d) or calcium ionophore (e,f). Cells were fixed and stained for β-tubulin. Scale bars 10 µm (a,b), (c,d), and (e,f). **(B)** Quantitative analysis of the frequency of microtubule protrusions. BMMCs incubated with various concentrations of miltefosine (5–15 µM) and BMMC controls were activated by FcεRI aggregation (antigen), thapsigargin, or calcium ionophore. Values indicate mean ± SD (*n* = 3); **p < 0.01 and ***p < 0.001. **(C)** Effect of miltefosine on the morphology of RPE1 cells. Cells incubated in the absence (a) or presence of 5 (b) and 15 µM miltefosine (c) were fixed and stained for β-tubulin. Scale bars 10 µm. **(D)** Chemotaxis assay. Migration tracks in control cells and cells treated with 15 µM miltefosine. Tracks from a representative experiment were aligned with their starting points at the coordinate position [0, 0]. Black tracks indicate individual cells with net migration toward the left chamber that contained Ag (100 ng/ml), red tracks indicate cells migrating in the opposite direction. Yellow crosses represent the average of endpoints. Representative experiments out of four repetitions are shown. **(E)** Cell migration. Migration velocities and accumulated distances of cells treated with 15 µM miltefosine relative to the control cells. Data are mean ± SD (*n* = 5); *p < 0.05.

Generation of protrusions containing microtubules is a characteristic feature of activated BMMCs attached to fibronectin (4). To evaluate the effect of miltefosine on generation of such protrusions, cells were pre-treated for 15 min with 15 µM miltefosine, and thereafter activated by Ag, thapsigargin, calcium ionophore, or pervanadate in the presence of miltefosine. Control cells activated by FcεRI aggregation using Ag (Figure 2Aa), thapsigargin (Figure 2Ac), calcium ionophore (Figure 2Ae), or pervanadate (data not shown) formed typical microtubule protrusions. On the other hand, cells activated in the presence of miltefosine by FcεRI aggregation using Ag (Figure 2Ab), thapsigargin (Figure 2Ad), calcium ionophore (Figure 2Af), or pervanadate (data not shown) were not capable to form microtubule protrusions. Statistical evaluation revealed a dose-dependent inhibitory effect of miltefosine on the formation of microtubule protrusions (Figure 2B). Activation of cells with Ag, thapsigargin, calcium ionophore, or pervanadate showed a similar response to miltefosine treatment, and already 5 µM miltefosine significantly inhibited formation of microtubule protrusions. Higher concentrations of miltefosine resulted in changes of cell morphology; cells were more spherical. Generation of microtubule protrusions was not affected when cells were activated by Ag in the presence of 15 µM MβCD. When cells were activated by Ag in the presence of 5 mM MβCD, the generation of protrusions reached 43 ± 14% (mean ± SD; *n* = 2) of the control, and in the presence of 10 mM MβCD, 3.4 ± 3% (mean ± SD; *n* = 2) of the control. Therefore, MβCD has to be used at substantially higher concentration to get a similar effect as miltefosine.

The activation with Ag, thapsigargin, or pervanadate was also performed in primary culture of BMMCs from BALB/c mice. Similarly as in the BMMC cell line, miltefosine inhibited generation of microtubule protrusions in these cells (data not shown). Miltefosine at concentration 5 µM did not distinctly affect microtubules in adherent RPE1 cells (Figure 2Ca,b). At higher miltefosine concentration (15 µM), cells started to round up (Figure 2Cc). These data document that miltefosine, even at low concentrations, strongly affects microtubule organization after cell activation both in BMMCs in the form of primary culture or cell line. On the other hand, 5 µM miltefosine did not affect microtubules in RPE1 or osteosarcoma U2OS cells (data not shown).

Ag-induced chemotactic response is essential for local accumulation of mast cells in the body, where they might perform their physiological roles. To study the effect of miltefosine on the migration of BMMCs to Ag, chemotaxis assays were performed. As shown in a representative experiment, miltefosine inhibited chemotaxis toward Ag. Moreover, tracks in miltefosine-treated cells were substantially shorter when compared to control cells (Figure 2D). The cell migration assay without chemoattractant revealed that both mean cell velocity (Figure 2E, left panel) and mean accumulated distance (Figure 2E, right panel) of all moving cells decreased in cells treated with 15 µM miltefosine. On the other hand, cell motility was not affected by 1 mM MβCD, and an inhibitory effect was observed only at 10 mM concentration of MβCD (data not shown). These data suggest that inhibition of Ag-induced chemotaxis by miltefosine is due to suppression of cell motility.

### Miltefosine Affects Tyrosine Phosphorylation and Aggregation of FcεRI Receptors in Activated BMMCs

**Figure 3:**
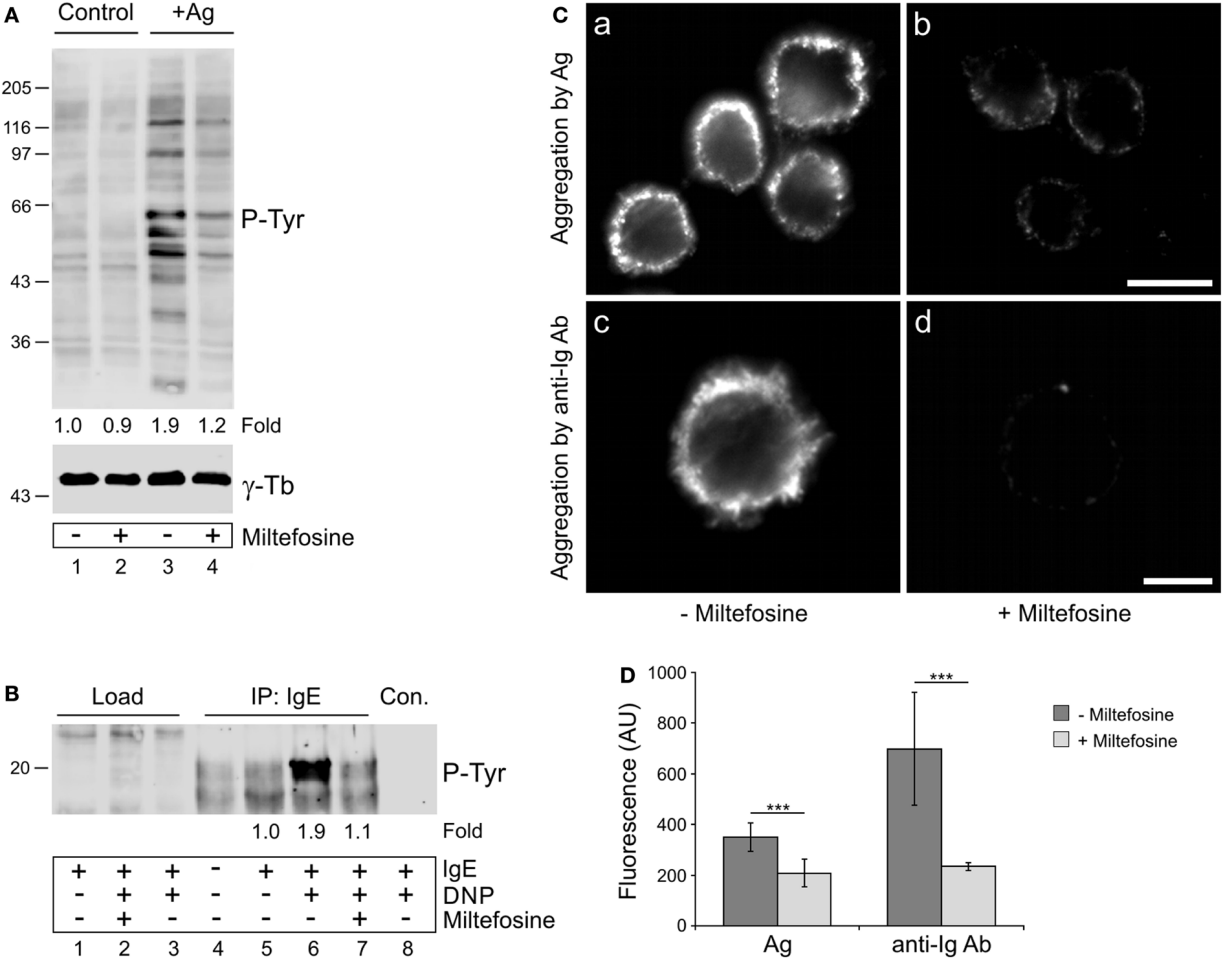
Miltefosine affects tyrosine phosphorylation and aggregation of high affinity IgE receptors (FcεRIs) in activated bone marrow-derived mast cells (BMMCs). **(A)** Comparison of protein tyrosine phosphorylation level (P-Tyr) in control cells and cells activated by FcεRI aggregation (+Ag) in the absence (lanes 1 and 3) or presence (lanes 2 and 4) of miltefosine. γ-Tubulin (γ-Tb) served as a loading control. Representative image out of three repetitions is shown. Numbers under the blot indicate relative amounts of P-Tyr normalized to control cells and to the amount of γ-tubulin in individual samples (fold). **(B)** Comparison of FcεRI receptor phosphorylation (P-Tyr) in the absence (lanes 1, 3–6, and 8) or presence (lanes 2 and 7) of miltefosine. Cells sensitized with mouse IgE to Ag were incubated with or without miltefosine, activated or not by Ag (DNP), and extracts were precipitated with anti-IgE Ab immobilized on protein A beads. In the control, protein A without Ab was incubated with the cell extract (lane 8, Con.). Note the difference in signal intensities in the positions of co-precipitated FcεRI receptors when cells were incubated without (lane 6) or with (lane 7) miltefosine. Representative image out of three repetitions is shown. Numbers under the blot indicate relative amounts of P-Tyr normalized to sensitized cells (fold). **(A,B)** Bars on the left indicate positions of molecular weight markers in kDa. **(C)** Comparison of FcεRI aggregation in the absence or presence of miltefosine. Cells sensitized with mouse IgE to Ag were incubated without (a,c) or with (b,d) miltefosine and activated by crosslinking of bound IgE with Ag (a,b; aggregation by Ag) or with anti-mouse Ig Ab conjugated with DY549 (c,d; aggregation by anti-Ig Ab). Cells were fixed with formaldehyde, and in the case of Ag-activated cells (a,b), stained with anti-mouse Ab conjugated with DY549. Images (a,b) and (c,d) were collected and processed under identical conditions. Scale bars, 10 µm (a,b); 5 µm (c,d). **(D)** Analysis of fluorescence intensity of FcεRI aggregation in the absence or presence of miltefosine. BMMCs were activated by crosslinking of bound IgE with Ag (Ag) or with anti-mouse Ig Ab (anti-Ig Ab). Values indicate mean ± SD (Ag, *n* = 30; anti-Ig Ab, *n* = 5); ***p < 0.001.

It has been reported that miltefosine, as a lipid raft modulator, could interfere with the structural organization of FcεRI receptors in activated mast cells and thus inhibit downstream signaling events (15). Because protein tyrosine phosphorylation plays an essential role in propagation of signals in BMMCs activated by FcεRI aggregation, we evaluated the overall protein tyrosine phosphorylation level (P-Tyr) in control and miltefosine pre-treated cells activated by Ag-mediated FcεRI aggregation. While the P-Tyr level increased in Ag-activated cells when compared to non-activated cell controls (Figure 3A, lane 3), it decreased in cells treated with miltefosine (Figure 3A, lane 4). It is well established that BMMC activation by Ag proceeds through tyrosine phosphorylation of immunoreceptor tyrosine-based activation motifs (ITAMs) located on the cytoplasmic tails of FcεRI β and γ subunits (33). We found that BMMCs activated with Ag showed a significantly increased level of P-Tyr on FcεRI (Figure 3B, lane 6) when compared with non-activated cells (Figure 3B, lanes 4–5). Phosphorylation of FcεRI in activated cells clearly decreased in the presence of miltefosine (Figure 3B, lane 7). Quantification of overall P-Tyr levels and FcεRI receptor P-Tyr levels in activated and miltefosine-treated cells is shown in Figures S1A,B in Supplementary Material. These findings were corroborated by immunofluorescence staining of IgE bound to FcεRI receptors. BMMCs sensitized with IgE and activated with Ag showed clear aggregation of the bound IgE (Figure 3Ca). The same results were obtained when the bound IgE was aggregated with anti-Ig Ab (Figure 3Cc). However, when the cells were pre-treated with miltefosine, aggregation with either Ag (Figure 3Cb) or anti-Ig Ab (Figure 3Cd) was substantially suppressed. Image analysis revealed that the staining intensity of miltefosine-treated cells activated by crosslinking of bound IgE by Ag or by anti-mouse Ig Ab was significantly lower when compared to control cells (Figure 3D). These data directly demonstrate that miltefosine inhibits aggregation and tyrosine phosphorylation of FcεRI receptors at the plasma membrane in activated BMMCs.

We also evaluated whether miltefosine affects overall P-Tyr levels of Lyn and Syk kinases and LAT adaptor protein that are known to be involved in early stages of FcεRI-mediated activation (1). P-Tyr levels were compared after immunoprecipitations from control and Ag-activated cells in the absence or presence of miltefosine. P-Tyr levels of Lyn kinase were comparable both in non-activated and Ag-activated cells (Figure S2A in Supplementary Material, IP: Lyn, lanes 2–3), as reported previously (34), and were not affected by miltefosine (Figure S2A in Supplementary Material, IP: Lyn, lane 4). Ag-activation increased comparably the P-Tyr levels of Syk kinase both in the presence or absence of miltefosine (Figure S2A in Supplementary Material, IP: Syk, lanes 3–4). Finally, the P-Tyr level of LAT increased in Ag-activated cells and was slightly attenuated in miltefosine-treated cells (Figure S2A in Supplementary Material, IP: LAT, lanes 3–4). These data suggest that inhibition of aggregation and tyrosine phosphorylation of FcεRI receptors by miltefosine does not substantially affect consecutive stages of signal transduction.

### Miltefosine Does Not Inhibit Ca^2+^ Influx in Activated BMMCs but Localizes to the Cellular Membranes and Cytosol

**Figure 4:**
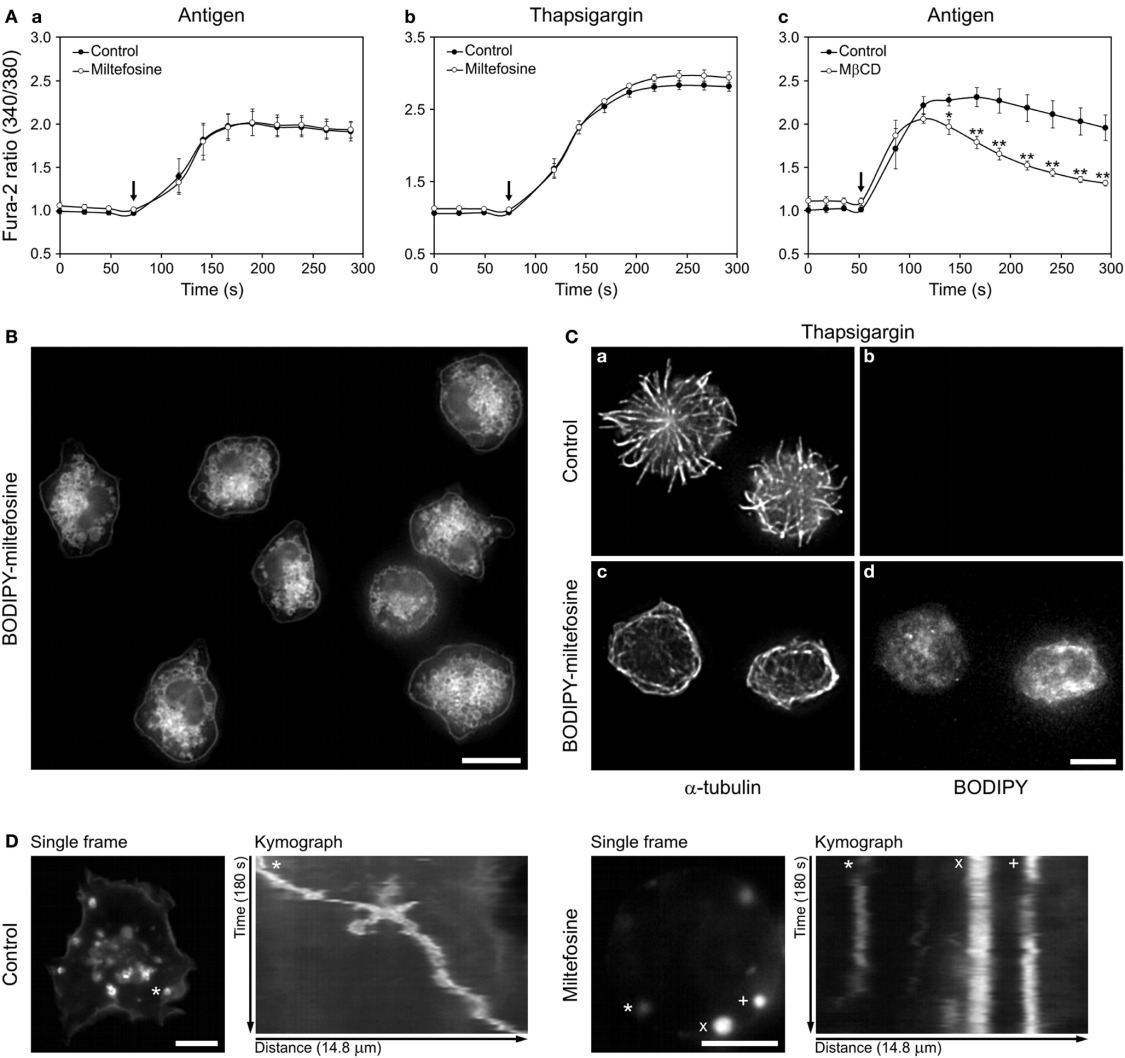
Miltefosine does not inhibit Ca^2+^ influx in activated bone marrow-derived mast cells (BMMCs), but localizes to cellular membranes and inhibits the granule movement. **(A)** Effect of miltefosine (a,b) and methyl-β-cyclodextrin (MβCD) (c) on intracellular Ca^2+^ level during cell activation. Sensitized cells were loaded with Fura-2-acetoxymethyl ester and pre-treated with or without (Control) miltefosine or MβCD. IgE-sensitized cells were activated by high affinity IgE receptor aggregation with Ag (a,c) or with thapsigargin (b). Arrows indicate addition of Ag or thapsigargin. Data represent mean ± SE [*n* = 3 for (a,b); *n* = 4 for (c)] from independent experiments performed in duplicates; *p < 0.05 and **p < 0.01. **(B)** BODIPY-miltefosine localizes to the cellular membranes and cytosol. Live-cell imaging of cells incubated with BODIPY-miltefosine. Scale bar, 10 µm. **(C)** BODIPY-miltefosine inhibits microtubule reorganization in activated cells. BMMCs treated or untreated (control) with BODIPY-miltefosine were activated by thapsigargin, fixed and stained for α-tubulin (a,c). Staining of BODIPY (b,d). Images (b,d) were collected and processed under identical conditions. Scale bar, 5 µm (a–d). **(D)** Time-lapse imaging of wheat germ agglutinin-stained intracellular granules in control and miltefosine-treated BMMCs. First frames from 180s time-lapse imaging and kymographs of stained granules are shown. The same track length (14.8 µm) was used for analysis in both cases. The tracked granules are marked by asterisks, cross, and diagonal cross.

We have reported that reorganization of microtubules in later stages of BMMC activation depends on Ca^2+^ influx (4). To test whether miltefosine affects the Ca^2+^ influx, we measured the level of intracellular Ca^2+^ after BMMC activation in control and miltefosine-treated cells (Figure 4A). Miltefosine did not inhibit the release of Ca^2+^ from the ER (data not shown) nor the influx of extracellular Ca^2+^ in cells activated with FcεRI aggregation using Ag (Figure 4Aa). Miltefosine slightly increased the Ca^2+^ influx in thapsigargin-activated cells (Figure 4Ab), and this trend was also observed in cells activated by calcium ionophore (data not shown). In contrast, treatment with 10 mM MβCD inhibited Ca^2+^ influx in Ag-activated BMMCs (Figure 4Ac). This points to the fact that these compounds, at concentrations causing similar inhibition of degranulation and formation of microtubule protrusions, have different effect on the signaling pathway leading to the Ca^2+^ influx in activated cells.

To evaluate the distribution of miltefosine in BMMCs we applied BODIPY-labeled miltefosine (28). Using this tool we localized it on the plasma membrane, intracellular membranous structures, as well as in the cytosol (Figure 4B). Internalization of BODIPY-miltefosine was rapid, as intracellular structures were decorated already after 1 min incubation with 15 µM BODIPY-miltefosine. When the cells were incubated with 1 µM BODIPY-miltefosine for 15–60 min, the intensity of staining increased in a time-dependent manner. BODIPY-miltefosine worked in the same way as the untagged miltefosine, as BODIPY-miltefosine inhibited generation of microtubule protrusions in cells activated with thapsigargin (Figure 4Cc,d; BODIPY-miltefosine), in contrast to control cells not treated with BODIPY-miltefosine (Figure 4Ca,b; control).

Secretory granules in living mast cells can be visualized by tagged WGA (5). Time-lapse imaging of BMMCs pre-treated with WGA-AF555 followed by kymograph analysis revealed that in comparison to control cells, 15 µM miltefosine inhibited movements of some granules (Figure 4D). On the other hand, 10 mM MβCD did not affect the granule movement (data not shown).

These data show that although miltefosine inhibits aggregation and phosphorylation of FcεRIs on the plasma membrane in activated cells, it does not inhibit the Ca^2+^ influx in activated cells. Moreover, miltefosine could affect signaling pathways in the cytosol as it rapidly localizes into the cell interior of BMMCs and influences granule movements.

### Miltefosine Inhibits Ca^2+^-Dependent PKCs in BMMCs

**Figure 5:**
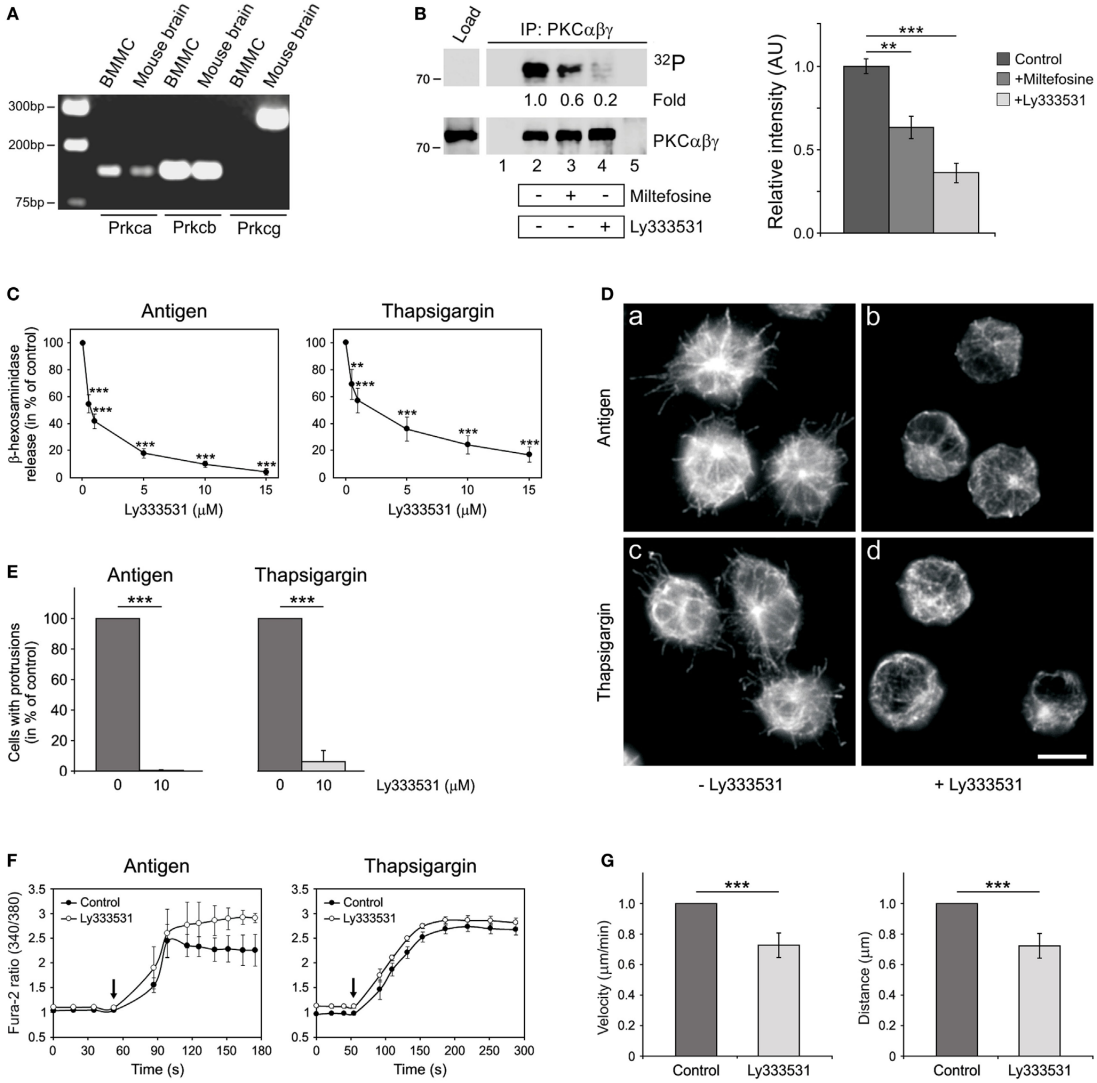
Miltefosine inhibits conventional protein kinase C (cPKC) activity in bone marrow-derived mast cells (BMMCs), and specific cPKC inhibitor Ly333531 affects degranulation, generation of microtubule protrusions, Ca^2+^-influx, and cell motility in a similar way as miltefosine. **(A)** Expression profile of cPKCs in BMMCs. Gel-based RT-PCR analysis of mouse PKCα (*Prkca*), PKCβ (*Prkcb*), and PKCγ (*Prkcg*). Mouse brain served as positive control. **(B)** The kinase activity in PKCαβγ immunocomplexes is inhibited by miltefosine. BMMC lysates were precipitated with anti-PKCαβγ Ab bound to protein A carrier. Immunocomplexes were subjected to *in vitro* kinase assay, electrophoretically separated, and detected by autoradiography (^32^P). The presence of PKCαβγ in immunocomplexes was confirmed by immunoblotting with anti-PKCαβγ Ab. (Left panel) Precipitation from resting cells (lane 2), cells pre-treated with 15 µM miltefosine (lane 3), and cells pre-treated with 10 µM Ly333531 (lane 4). Controls contained protein A with Ab (lane 1) and protein A without Ab incubated with the cell extract (lane 5). Bars on the left indicate positions of molecular weight markers in kDa. Representative image out of three repetitions is shown. Numbers under the blot indicate relative amounts of phosphorylated cPKCs normalized to control cells and to the amount of precipitated cPKCs in individual samples (fold). (Right panel) Quantification of autoradiographs by densitometry. Control untreated cells or cells pre-treated with 15 µM miltefosine (+miltefosine) or 10 µM Ly333531 (+Ly333531). Relative intensities of phosphorylated cPKCs normalized to control cells and to the amount of precipitated cPKCs in individual samples. Data represent mean ± SD (*n* = 3); **p < 0.01 and ***p < 0.001. **(C)** cPKCs are essential for BMMC degranulation. BMMCs pre-incubated with different concentrations of Ly333531 (0.5–15 µM) were activated by Ag or thapsigargin and degranulation was measured by β-hexosaminidase release. Values represent mean ± SD (*n* = 4); **p < 0.01 and ***p < 0.001. **(D)** cPKCs regulate microtubule organization. BMMCs incubated in the absence (a,c) or presence (b,d) of 10 µM Ly333531 were activated by high affinity IgE receptor (FcεRI) aggregation (a–b; antigen) or thapsigargin (c–d). Cells were fixed and stained for β-tubulin. Scale bar 10 µm (a–d). **(E)** Quantitative analysis of the frequency of microtubule protrusions. BMMCs incubated with 10 µM Ly333531 and BMMC controls were activated by FcεRI aggregation (antigen) or thapsigargin. Values indicate mean ± SD (*n* = 3); ***p < 0.001. **(F)** cPKCs do not inhibit Ca^2+^ influx in activated BMMCs. Sensitized cells were loaded with Fura-2-acetoxymethyl ester, pre-treated without (control) or with 10 µM Ly333531, and activated by Ag or thapsigargin. Arrows indicate addition of Ag or thapsigargin. Data represent mean ± SE (*n* = 2 for Ag; *n* = 3 for thapsigargin) of independent experiments performed in duplicates. **(G)** Cell migration depends on cPKCs. Migration velocities and accumulated distances of cells treated with 10 µM Ly333531 relative to the control cells. Data are mean ± SD (*n* = 3); ***p < 0.001.

It is well established that Ca^2+^ and diacylglycerol-regulated conventional PKCs (cPKCs) are important for mast cell degranulation (35). As miltefosine was reported to inhibit PKC in mouse NIH/3T3 cells (20), we evaluated the possibility that miltefosine could affect the activity of cPKCs in BMMCs. There are four isoforms of cPKCs, specifically, PKCα, PKCβI, PKCβII, and PKCγ (36). The expression of cPKCs in BMMCs was determined by a gel-based RT-PCR analysis using mouse brain as a positive control. We found that PKCβ isoforms were the most prominent, while PKCγ was under the detection limit (Figure 5A).

*In vitro* kinase assay revealed that miltefosine can inhibit the activities of cPKCs immunoprecipitated from BMMCs (Figure 5B left panel, lane 3). As a positive control we used Ly333531 inhibitor, which efficiently inhibited autophosphorylation of cPKCs immunoprecipitated from BMMCs (Figure 5B left panel, lane 4). Quantification of cPKC phosphorylation levels is shown in Figure 5B right panel. Ly333531 preferentially inhibits PKCβ isoforms. The IC_50_ values for PKCβI and PKCβII are 4.7 and 5.9 nM, respectively, while for PKCη, -δ, -γ, -α, -ε, and -ζ are 0.052, 0.25, 0.30, 0.36, 0.6, and >100 μM, respectively (37). As PKCβ isoforms were the most abundant in BMMCs, we examined the effect of Ly333531 on degranulation, generation of microtubule protrusions, Ca^2+^ influx, and cell migration.

Ly333531, similarly as miltefosine, significantly inhibited β-hexosaminidase release in a dose-dependent manner in cells activated by FcεRI aggregation (Figure 5C, antigen), thapsigargin (Figure 5C), or pervanadate (data not shown). Generation of microtubule protrusions was attenuated by Ly333531 treatment in BMMCs activated by Ag or thapsigargin (Figure 5D). Statistical evaluation of this effect is shown in Figure 5E. While Ly333531 did not affect the Ca^2+^ influx (Figure 5F), cell motility was inhibited by its treatment (Figure 5G). Collectively, these data demonstrate that cPKC inhibitor Ly333531 modulates mast cell functions in the same way as miltefosine. This suggests that miltefosine might regulate these processes through the inhibition of cPKC activity.

## Discussion

Mast cell activation by crosslinking of FcεRIs triggers the signaling pathways resulting in Ca^2+^ influx, degranulation, and synthesis of new mediators. Pharmaceutical agents that modulate integrity of the membrane environment or affect mast cell signaling events might be potentially used as treatments for mast cell-driven diseases. A promising candidate is the lipid raft modulator miltefosine (15). The obvious advantages of miltefosine are known side effects, which are relatively safe, dose-dependent, and reversible (38). Clinical application has been limited to topical and oral treatments, and among major known side effects belong loss of appetite, vomiting, nausea, and diarrhea after long oral treatment of high daily dosages (150 mg and higher) (39). Although miltefosine was approved for the treatment of various diseases (40), the molecular mechanism of its action in mast cells remains poorly understood.

In human skin mast cells, it has been suggested that miltefosine affects organization of FcεRIs in the plasma membrane, which then leads to modulation of subsequent activation steps (7, 15). Here we used murine BMMCs as they are well responsive to both allergic and non-allergic stimuli and they are generally used for *in vitro* studies, as they can be easily produced in large amounts (41). Different murine mast cell types, specifically connective tissue mast cells (CTMCs), mucosal tissue mast cells (MMCs), and BMMCs, all express c-kit and FcεRIs on their surfaces, and can degranulate upon Ag-activation (42). CTMCs and BMMCs differ in TLR (toll-like receptor)-induced cytokine and chemokine production, expression of STAT proteins, and response to IL-18 (42, 43). However, such differences should not have effect on early signaling events after FcεRI aggregation in these cells. Molecular mechanisms of miltefosine action in early stages of Ag-activation could be, therefore, similar both in BMMCs and CTMCs (e.g., human skin mast cells).

Our results demonstrate that BMMCs have similar sensitivity to miltefosine as human mast cells (7) and that 15 µM miltefosine does not change cell viability. Degranulation was attenuated in miltefosine-treated cells in a dose-dependent manner after cell activation by FcεRI aggregation, thapsigargin, pervanadate, or calcium ionophore. Obtained data were comparable to the mediator release of miltefosine-treated human primary mast cells activated by Ag (7). We have previously shown that activation of BMMCs leads to rapid cytoskeleton rearrangement and generation of microtubule protrusions (4). Here, we demonstrate that in activated BMMCs, miltefosine suppresses formation of these protrusions and affects cell morphology more effectively than in other tested cell types. As miltefosine inhibited formation of protrusions containing microtubules more effectively than degranulation, we suppose that physical integration of miltefosine into the plasma membrane contributes to this effect. Moreover, miltefosine also inhibits chemotaxis to Ag and cell motility, which points to a limited capability of miltefosine-treated cells to accumulate in the site of disease manifestation. These data indicate that miltefosine could modulate physiology of mast cells at different levels.

To get deeper insight into the effect(s) of miltefosine in mast cells, we evaluated early steps of cell activation after aggregation of FcεRIs by Ag. Miltefosine inhibited both the overall tyrosine phosphorylation level and the aggregation and tyrosine phosphorylation of FcεRIs. Diminished FcεRIs phosphorylation could be due to the changes in plasma membrane properties that inhibit formation of large FcεRI aggregates. Protein tyrosine kinase Lyn can, therefore, only partially phosphorylate both ITAMs, located on the cytoplasmic tails of FcεRI β and γ subunits (33). Our data thus support the previous suggestion that miltefosine attenuates FcεRI-mediated signaling events at the plasma membrane (7, 15).

Miltefosine treatment, however, did not affect overall P-Tyr levels of Lyn and Syk kinases and only slightly diminished P-Tyr level of adaptor protein LAT in Ag-activated cells. Moreover, miltefosine did not inhibit the release of Ca^2+^ from ER nor the extracellular Ca^2+^ influx. This indicates that the low level of FcεRI phosphorylation observed after cell activation in the presence of miltefosine is sufficient to activate the subsequent signaling cascade. It was reported previously that the formation of large FcεRI aggregates is not necessary for triggering the signaling responses, and that Ag-activated mast cells propagate signals from small signaling domains formed around dimerized FcεRIs (44). Moreover, it is well established that cholesterol-dependent ordered lipids regulate the Ca^2+^ channel (Orai1), and polyunsaturated fatty acids with phosphoinositides regulate coupling of Orai1 to ER Ca^2+^ sensor (STIM1) in SOCE (45). Our results suggest that although miltefosine affects the membrane composition and aggregation of FcεRI receptors, this is not reflected in the inhibition of the Ca^2+^ influx level through SOCE.

Calcium mobilization has a critical impact on activation of many signal-transducing proteins that are involved in the regulation of mast cell degranulation. We observed differential miltefosine inhibitory effects on degranulation in BMMCs activated by Ag, thapsigargin, pervanadate, or calcium ionophore. This probably reflects the specificity and site(s) of action of the used activators. When miltefosine was compared with MβCD, a typical lipid raft disruptor, MβCD had to be used at ~1,000-fold higher concentrations to inhibit degranulation, microtubule rearrangement, and cell motility to the levels observed in miltefosine-treated cells. Moreover, MβCD effectively inhibited Ca^2+^ influx in activated BMMCs. The fact that miltefosine does not inhibit the Ca^2+^ influx indicates that it could attenuate degranulation by affecting the cytosolic signaling pathway(s) after SOCE. It is well established that Ca^2+^-dependent PKCs are important for mast cell degranulation (35). In BMMCs, we detected PKCα and PKCβ isoforms, and their activities were inhibited by miltefosine. Similarly as miltefosine, cPKC inhibitor Ly333531 inhibited mast cell degranulation, generation of microtubule protrusions and cell migration, while Ca^2+^ influx was not affected. At present, there is a search for new compounds that inhibit PKCs. It has been proposed that drugs modulating the activity of PKCs could have a major impact on the treatment of immune disorders (46). Deciphering the exact mechanism of PKC inhibition by miltefosine warrants further investigation.

In BMMCs, miltefosine localized to the plasma membrane, cytosol, and intracellular membranous structures including granules. Staining of intracellular structures with BODIPY-miltefosine was previously shown in *Leishmania donovani* at 7 µM concentration for 4 h (28). Here, we show that internalization of BODIPY-miltefosine in BMMCs is fast and even at 1 µM concentration it incorporates to intracellular membranous structures within 15 min. Moreover, cytosolic miltefosine attenuated movement of some intracellular BMMC granules.

It was reported that miltefosine inhibits PI3K/Akt survival pathway in tumor (17) and skeletal muscle cells (47). As PI3K/Akt pathway is important for survival, growth, and differentiation in activated mast cells (48), miltefosine might affect also these activities. It is known that Akt is partially activated by phosphorylation at Thr^308^ through PI3K/PDK1 (3-phosphoinositide-dependent protein kinase 1) pathway and fully activated by additional phosphorylation at Ser^473^ through PI3K/mTORC2 (mechanistic target of rapamycin complex 2) pathway (49). We have found that miltefosine in Ag-activated BMMCs inhibited phosphorylation on both Thr^308^ and Ser^473^ (Figure S2B in Supplementary Material, lanes 2–3). As miltefosine enters to cytosol, it could affect Akt-dependent cellular activities that will manifest in later stages of mast cell activation.

Effective treatments of mast cell-derived mastocytosis (8), urticaria (9), and atopic dermatitis (10) were after topical application of miltefosine. On the other hand, miltefosine side effects in gastrointestinal tract were observed after oral use in cancer patients. We do not assume that these side effects could be explained by inhibition of degranulation by miltefosine, as vomiting and diarrhea are connected with increased number of degranulating mast cells in digestive tract (50). PKCs are central kinases and their inhibition by miltefosine could also affect other cell types. However, sensitivity of cells to miltefosine can substantially differ as we documented on changes in microtubule organization in BMMCs or RPE1 and U2OS cells.

Based on the published data and findings in this report, we suggest that miltefosine might affect mast cell activation by different mechanisms. First, incorporation of miltefosine into the plasma membrane influences its properties. This results in morphological changes, inhibition of FcεRI aggregation by Ag, chemotaxis, and generation of microtubule protrusions in the course of specific (Ag) or unspecific (thapsigargin, pervanadate, and calcium ionophore) activation. Second, as miltefosine does not inhibit Ca^2+^ response and rapidly enters into the cytosol, it is able to modulate the intracellular signaling pathways important for degranulation. Upon cell activation, Ca^2+^ and PKCs act to reverse the inhibitory mechanisms of granule fusion and activate proteins and cellular events to promote the granule fusion (51). Because miltefosine inhibits Ca^2+^-dependent cPKCs, it could interfere with this pathway. Third, miltefosine attenuates movements of intracellular granules. It could, therefore, affect the function of microtubule motors that are important for transport of secretory granules in mast cells (5).

In conclusion, our data suggest that miltefosine modulates BMMCs both at the plasma membrane and in the cytosol by an inhibition of Ca^2+^-dependent PKCs. This leads to substantial morphological changes, inhibition of chemotaxis and degranulation. Effective treatment of mast cell-derived diseases by miltefosine could be, therefore, based on its action at multiple sites in the cells.

## Data Availability Statement

The raw data supporting the conclusions of this manuscript will be made available by the authors, without undue reservation, to any qualified researcher.

## Author Contributions

ZR designed, performed and analyzed microscopic experiments, and prepared the manuscript. VS performed immunoprecipitation and kinase assays. TP performed intracellular Ca^2+^ measurements and PD planned the experiments, helped with result interpretation, and revised the manuscript. All authors approved the final version of the manuscript.

## Conflict of Interest Statement

The authors declare that the research was conducted in the absence of any commercial or financial relationships that could be construed as a potential conflict of interest.

## Abbreviations

Ab(s), antibody(ies); Ag, antigen; BMMC, bone marrow-derived mast cell; BSA, bovine serum albumin; DNP, dinitrophenyl; FcεRI, high affinity IgE receptor; Fura-2-AM, Fura-2-acetoxymethyl ester; HRP, horseradish peroxidase; ITAMs, immunoreceptor tyrosine-based activation motifs; MβCD, methyl-β-cyclodextrin; mAb, monoclonal antibody; 4-NAG, 4-nitrophenyl N-acetyl-β-D-glucosaminide; cPKCs, Ca^2+^- and diacylglycerol-regulated conventional protein kinases; PI3K, phosphatidylinositol 3-kinase; SOCE, store-operated Ca^2+^ entry; WGA, wheat germ agglutinin.
