# Supplementary material for: Mast Cell Activation and Microtubule Organization Are Modulated by Miltefosine Through Protein Kinase C Inhibition
Source: Front Immunol. 2018 Jul 9;9:1563. doi: 10.3389/fimmu.2018.01563 (PMC6046399; doi:10.3389/fimmu.2018.01563)
Supplement: Supplementary file 1 [file presentation_1.PDF]

Supplementary Material

Mast Cell Activation and Microtubule Organization is Modulated by Miltefosine through Protein Kinase C Inhibition

Zuzana Rubíková, Vadym Sulimenko, Tomáš Paulenda and Pavel Dráber*

*** Correspondence:** Dr. Pavel Dráber: paveldra@img.cas.cz

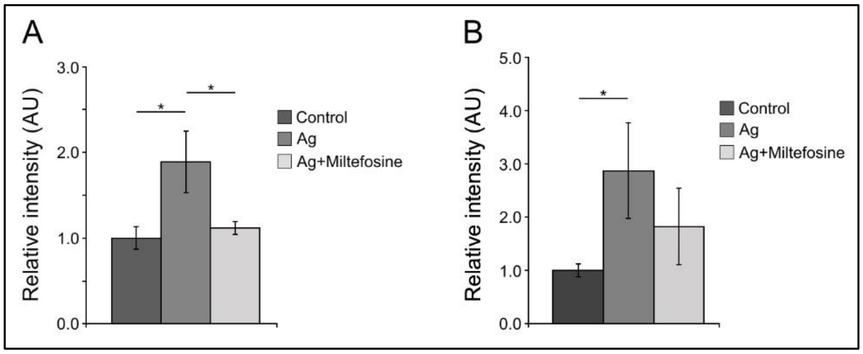

Figure S1. Quantitative analysis of tyrosine phosphorylation in miltefosine-treated and activated BMMCs.

Control miltefosine-untreated cells and cells activated by FcεRI aggregation in the absence (Ag) or presence of miltefosine (Ag+Miltefosine). ***(A)*** Densitometric quantification of overall protein tyrosine phosphorylation level shown in Fig. 3*A*. Relative intensities of P-Tyr normalized to control cells and to the amount of γ-tubulin in individual samples ***(B)*** Densitometric quantification of FcεRI receptor phosphorylation level shown in Fig. 3*B*. Relative intensities of P-Tyr normalized to control cells. ***(A-B)*** Data represent means ± SD (n=3); $*p < 0.05$.

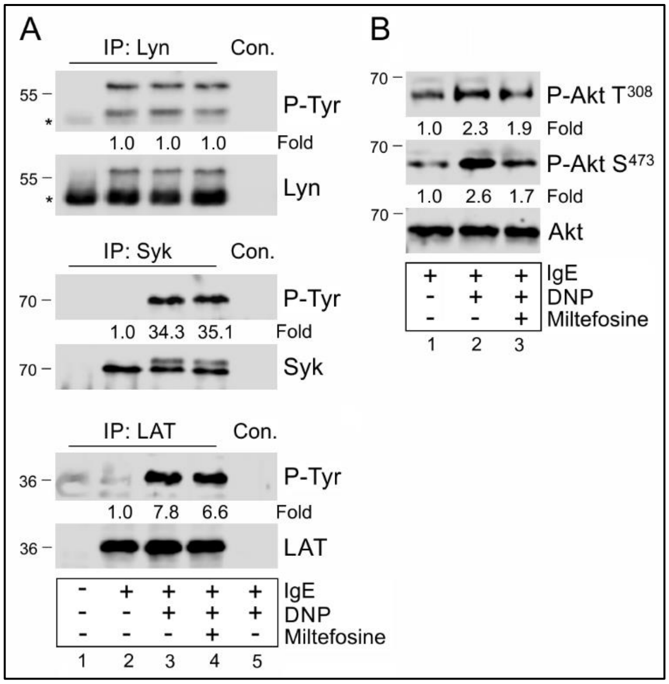

Figure S2. Effect of miltefosine on early activation events in mast cells.

(A) Comparison of Lyn, Syk and LAT phosphorylation levels (P-Tyr) in control sensitized cells (lane 2) and cells activated by FcεRI aggregation (lanes 3-5) in the absence (lanes 1-3, 5) or presence (lane 4) of miltefosine. Cells sensitized with mouse IgE to Ag were incubated with or without miltefosine, activated or not by Ag (DNP), and extracts were precipitated with Abs to Lyn, Syk or LAT immobilized on protein A beads. Immobilized Abs not incubated with cell extracts (lane 1). Protein A without Abs, incubated with the cell extract (lane 5, Con.). Representative images out of two repetitions are shown. Numbers under the blot indicate relative amounts of P-Tyr normalized to sensitized cells and to the amount of Lyn, Syk or LAT in individual samples (Fold). Asterisks denote positons of heavy chain of Ab. ***(B)*** Comparison of Akt phosphorylation levels in control sensitized cells (lane 1) and cells activated by FcεRI aggregation (lanes 2-3) in the absence (lanes 1-2) or presence (lane 3) of miltefosine. Whole cell lysates were probed with Abs to p-Akt (Thr^{308}) and p-Akt (Ser^{473}). Akt served as a loading control. Representative image out of two repetitions is shown. Numbers under the blot indicate relative amounts of p-Akt (Thr^{308}) and p-Akt (Ser^{473}) normalized to control cells and to the amount of Akt in individual samples (Fold). ***(A-B)*** Bars on the left indicate positions of molecular weight markers in kDa.
